# Metagenomics profiling of the microbial community and functional differences in solid-state fermentation vinegar starter (seed *Pei*) from different Chinese regions

**DOI:** 10.3389/fmicb.2024.1389737

**Published:** 2024-05-02

**Authors:** Dong Han, Yunsong Yang, Zhantong Guo, Ken Chen, Shuwen Dai, Yuanyuan Zhu, Yuqin Wang, Zhen Yu, Ke Wang, Peng Liu, Chunchi Rong, Yongjian Yu

**Affiliations:** ^1^School of Grain Science and Technology, Jiangsu University of Science and Technology, Zhenjiang, China; ^2^Jiangsu Provincial Engineering Research Center of Grain Bioprocessing, Jiangsu University of Science and Technology, Zhenjiang, China

**Keywords:** metagenomic features, microbial diversity and function, microbiome, seed *Pei*, solid-state fermentation vinegar

## Abstract

**Introduction:**

The starter used in solid-state fermentation (SSF) vinegar, known as seed *Pei* is a microbial inoculant from the previous batch that is utilized during the acetic acid fermentation stage. The seed *Pei*, which has a notable impact on vinegar fermentation and flavor, is under-researched with comparative studies on microorganisms.

**Methods:**

Herein metagenomics was employed to reveal the microbes and their potential metabolic functions of four seed *Pei* from three regions in China.

**Results:**

The predominant microbial taxa in all four starters were bacteria, followed by viruses, eukaryotes, and archaea, with *Lactobacillus* sp. or *Acetobacter* sp. as main functional taxa. The seed *Pei* used in Shanxi aged vinegar (SAV) and Sichuan bran vinegar (SBV) exhibited a higher similarity in microbial composition and distribution of functional genes, while those used in two Zhenjiang aromatic vinegar (ZAV) differed significantly. Redundancy analysis (RDA) of physicochemical factors and microbial communities indicated that moisture content, pH, and reducing sugar content are significant factors influencing microbial distribution. Moreover, seven metagenome-assembled genomes (MAGs) that could potentially represent novel species were identified.

**Conclusions:**

There are distinctions in the microbiome and functional genes among different seed *Pei*. The vinegar starters were rich in genes related to carbohydrate metabolism. This research provides a new perspective on formulating vinegar fermentation starters and developing commercial fermentation agents for vinegar production.

## Graphical Abstract

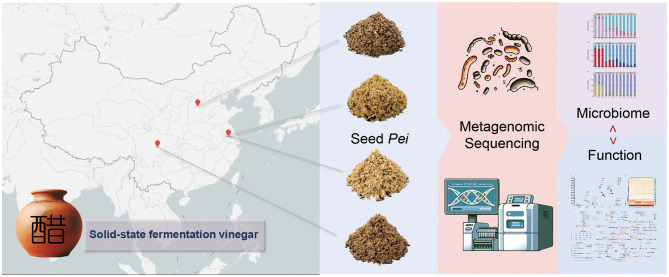

## 1 Introduction

Vinegar is an essential sour condiment in diets worldwide. Traditional vinegar brewing in China has predominantly favored solid-state fermentation (SSF) (Liu et al., 2004; Nie et al., 2017), by using grain-based substrates. This technique yields a richer flavor in vinegar than liquid-state fermentation, despite its controllability and struggles to match (Liu et al., 2018). Compared to the liquid-state fermentation of rice vinegar, SSF generally results in a higher production of compounds derived from microbial metabolism (Li et al., 2015). Renowned Chinese vinegars such as Zhenjiang aromatic vinegar (ZAV), Shanxi aged vinegar (SAV), and Sichuan bran vinegar (SBV) are all produced by SSF, although their processes are uniquely tailored (Hu et al., 2023). These vinegars use an open fermentation process with grains (rice, sorghum, wheat bran, or malt) as the main raw materials (Ho et al., 2017). The process involves multiple stages of saccharification, alcohol fermentation, and acetic acid fermentation and typically takes nearly 1 month to complete. ZAV is derived from glutinous rice, while SAV is derived from sorghum (Xia et al., 2019); both are steamed and fermented in two distinct pools. In contrast, SBV, made from raw wheat bran (Zhu et al., 2021), is completely fermented in a single tank. In the entire process, most of the raw materials are not sterilized (Wu et al., 2012). *Daqu* is used as the starter for both saccharification and alcohol fermentation. During acetic acid fermentation, vinegar *Pei* from the previous batch is used as a starter (Chen et al., 2015; Nie et al., 2015; Wang et al., 2016). In traditional food fermentation processes, it is a common practice to inoculate fresh substrates with solid or semi-solid cultures from the previous batch to initiate fermentation or modulate the ecological niche. Thus, for vinegar brewing, vinegar *Pei* represents both an intermediate stage in the fermentation process and a starter. Due to the variability in raw materials and the fermentation volume of different vinegar types, the actual fermentation times of seed *Pei* are primarily chosen based on experience.

The microorganisms used in fermentation are key in conferring unique flavors and nutritional value to fermented foods, with the starter studies on microbial communities across different SSF vinegar starter cultures, ensuring a smooth start of the fermentation process (Li et al., 2015). Due to its unique process characteristics, the seed *Pei* represents a self-assembled and stable microbial community (Li et al., 2016b; Lu et al., 2018). The microbial community succession occurs during each batch of fermentation, resulting in the production of substances that impart flavor to vinegar through microbial metabolism (Wang et al., 2016). Although extensive studies have elucidated microbial succession and its correlation with the synthesis of flavor compounds during a particular vinegar fermentation (Li et al., 2016b, 2024; Huang et al., 2022), there is a lack of comparative studies on microbial communities across different SSF vinegar staters. Isolating and culturing microorganisms from these substrates pose significant challenges as they are difficult to grow on existing culture media (Milanović et al., 2018). Additionally, these bacterial colonies can exist in a viable but non-culturable (VBNC) state, which can sometimes affect the results of culture-dependent methods (Vegas et al., 2013). To overcome this limitation, culture-independent methods such as denaturing gradient gel electrophoresis (PCR-DGGE), amplicon sequencing, and metagenomics have been the mainstay methodologies in investigating microbial communities. Metagenomics, in particular, offers an unprecedented resolution by quantifying the microbial relative abundance to a more detailed taxonomy, elucidating functional gene landscapes, and potentially uncovering novel groups that have not been cultivated (Zhang et al., 2021). It has become an instrumental technology in decoding the microbial and functional landscapes of fermented foods such as vinegar, *Daqu*, and fermented vegetables (Li et al., 2016b; Yang et al., 2021; Liu et al., 2023).

In this study, we focus on the acetic acid fermentation of SSF vinegar, using metagenomic sequencing to gain an insight into the composition and functionality of microbiomes within seed *Pei*. Furthermore, our research aims to identify marker microorganisms that are indicative of the vinegar starter cultures for various types of vinegar. We collected vinegar *Pei* from three different regional products (ZAV, SAV, and SBV) originating from four separate enterprises. First, we used metagenomic analysis of the seed *Pei* to decode its microbial constituency and functional characteristics. Second, we measured some physicochemical properties of the seed *Pei* and conducted correlation analyses with microbial species. Finally, metagenome-assembled genomes (MAGs) originating from disparate grains were comparatively analyzed. The findings are significant for elucidating regional variations in vinegar starter cultures and providing a knowledge base that could spearhead advancements in vinegar fermentation techniques.

## 2 Materials and methods

### 2.1 Sample collection

Each sample was produced by SSF during July to August 2023. ZAVa and ZAVb were collected from two vinegar manufacturers in Zhenjiang, Jiangsu Province, China (32.10 N 119.48 E, 32.12 N 119.46 E, respectively). They were both collected on the sixth day of fermentation. SAV was collected from Taiyuan, Shanxi Province, China (37.76 N 112.68 E) on the second day of fermentation. SBV was obtained from Meishan, Sichuan Province, China (30.20 N 103.81 E) on the tenth day of fermentation. ZAVa, ZAVb, and SBV were collected from the long fermentation tank. Each sample was taken from the top to the bottom from the front, center, and back of the tank and then mixed into one sample. SAV was produced in the fermentation vat, and the sample was taken from the center and the surrounding area, from the top to the bottom, and then mixed as one sample. Three replicates were taken from a mixture of three fermentation resources. Samples were placed in sterile bags and sealed and stored in a cooled environment. They were then transferred to the laboratory within 24 h for subsequent preservation at −80°C.

### 2.2 Physicochemical analysis

The water content was determined using the gravimetric approach, where vinegar *Pei* were subjected to desiccation at 105°C until they reached a stable mass. The water content was quantified by the proportion of dry to wet mass. The pH value, total acid, acetic acid, lactic acid, and reducing sugar content were analyzed as follows: 10 g seed *Pei* was mixed with 30 mL of deionized water and agitated at room temperature with a rotational speed of 100 rpm for 3 h. The filtrate was collected for measurement. The pH value was measured with a pH meter (Mettler-Toledo, FE28). The total acid content was gauged by neutralization titration using NaOH (Huang et al., 2022). The acetic acid and lactic acid analysis were carried out by the modifying method (Danova et al., 2023) using high-performance liquid chromatography (HPLC) Agilent 1260 Series (Agilent Technologies). Specifically, 2.5 mL of the filtrate was added with 1 mL of 106 g/L potassium ferricyanide solution and 1 mL of 300 g/L zinc sulfate solution sequentially. The mixture was left to sit for 1 h at room temperature. Before injection, the mixture was centrifuged and filtered through a 0.22-μm filter. The samples were analyzed on an Aminex HPX-87C Column (300 × 7.8 mm, Bio-Rad) held at 55°C, using 0.005 mol/L H_2_SO_4_ as the mobile phase at a flow rate of 0.6 mL/min. The reduced sugar content was determined through the application of the 3,5-dinitrosalicylic acid (DNS) assay (Huang et al., 2022). For the measurement of starch and total protein content, the seed *Pei* samples were first dried. The total protein content was determined using the spectrophotometric method according to the National Standards of the People's Republic of China GB/T 5009.5-2016 (Zhang et al., 2022). The starch content was determined according to the National Standards of the People's Republic of China GB/T 5009.9-2003, with specific procedures referenced from a previous study (Liu et al., 2020). All measurements were performed in triplicate.

### 2.3 DNA extraction, sequencing, and assembly

The DNA was extracted from *Pei* using the cetyltrimethylammonium bromide (CTAB) method, as previously reported (Huang et al., 2022). The quality and quantity of the extracted DNA were assessed by using a NanoDrop 2000 spectrophotometer (Thermo Scientific, USA) and agarose gel electrophoresis. The qualified DNA (> 50 ng/mL, OD_260_/OD_280_ 1.8~2.2, OD_260_/OD_230_ 1.8~2.2) was sent to Beijing Novozymes Technology Co. for metagenomic sequencing. The genomic DNA was randomly fragmented into 350 bp using ultrasonic disruption. The sequencing libraries were prepared through processes including end repair, A-tailing, adapter ligation, and purification. Libraries were sequenced by using the Illumina PE150 platform (Illumina, USA). More than 10 Gb of raw data were obtained for each sample sequencing. Raw data were filtered by Readfq (https://github.com/cjfields/readfq) to obtain clean data. The reads with low-quality bases (quality ≤ 38), or N bases reaching 10 bp, or overlaps with adapters exceeding 15 bp were removed. Furthermore, the reads were aligned to plant genomes by Bowtie2 software to remove potential host contamination (Langmead et al., 2018). Sequence assembly was performed by MEGAHIT software (Li et al., 2016a) with clean data, and sequences with lengths >500 bp were retained.

### 2.4 Annotation of microbial species, functional genes, and metabolic pathways

Open reading frame (ORF) prediction of assembled contigs was done using MetaGeneMark (http://topaz.gatech.edu/GeneMark/). De-redundancy of predicted ORFs was performed by CD-HIT software (http://www.bioinformatics.org/cd-hit/). The non-redundant genes obtained through this process are referred to as unigenes. Bowtie2 (Langmead et al., 2018) was used to annotate the abundance of unigenes in reads of clean data. Unigenes were aligned to NCBI's NR database, KEGG database, and Carbohydrate-Active enZYmes (CAZy) database using DIAMOND software (Buchfink et al., 2015) to obtain taxonomic annotations and functional annotations. For taxonomic annotations, each sequence was determined by the LCA algorithm of MEGAN software (Huson et al., 2016) selected from the results with alignment e-value ≤ 10 × minimum e-value. Species abundance and functional gene abundance were determined based on the results of annotation and the abundance of unigenes, respectively.

### 2.5 Metagenomic assembled genome (MAG) assembly and taxonomy annotation

The clean data of three repetitions in each sample were mixed and assembled into contigs using the default parameters of MEGAHIT (Li et al., 2016a). Each sample sequencing result (~30 Gb of clean data in total) was divided into different bins using MetaBAT 2 (Kang et al., 2019). Each bin was evaluated for quality by CheckM (Parks et al., 2015). Bins with ≥ 80% integrity and ≤ 10% contamination were considered as high-quality MAGs. The taxonomy annotation of high-quality bins was performed using the Genome Taxonomy Database Toolkit (GTDB-Tk) (Chaumeil et al., 2020). If the MAGs were not assigned at the species level by GTDB-Tk, they were defined as novel species. MAGs were phylogenetically analyzed using GTDB-Tk to identify 122 archaeal and 120 bacterial marker genes, and the phylogenetic tree was constructed using IQ-TREE2 (Minh et al., 2020). The tvBOT tool was used for phylogenetic tree visualization (Xie et al., 2023).

### 2.6 Data analysis and visualization

The Venn diagram illustrating species composition was generated using the online platform available at http://www.ehbio.com/test/venn/#/ (Chen et al., 2021). Alpha diversity was assessed using the vegan package (version 2.5-2) in R software, which can be accessed at https://cran.r-project.org/web/packages/vegan/index.html. Statistical evaluation was conducted using SPSS software, version 23.0, through a two-tailed *t*-test, with significance determined at a *p* < 0.05. Principal coordinate analysis (PCoA) was performed based on Bray–Curtis dissimilarity at the species level, with plotting performed using the principal coordinate combinations that contributed the most variance. The visualization of KEGG functional genes distribution and clustering tree of different samples was done based on Bray–Curtis distance using R software. Differences across the taxonomic levels within seed *Pei* were investigated using the linear discriminant analysis (LDA) effect size (LEfSe) method (Segata et al., 2011). The correlation between microbial species in seed *Pei* was calculated using the iNAP web tool (Feng et al., 2022). The top 1,000 species in abundance were analyzed using Spearman's rank correlation. Only correlations with correlation coefficients | r | > 0.8 and *p* < 0.01 were used to construct the co-occurrence network. The co-occurrence network was visualized using Gephi software (version 0.10.1) (Bastian et al., 2009). As for CAZymes, quantitative differential graphs, heatmaps, and MAG quality distribution charts were generated using the online visualization website https://www.chiplot.online/. Redundancy analysis (RDA) was conducted using the online analysis tool available at http://www.cloud.biomicroclass.com/CloudPlatform/SoftPage/CCA.

## 3 Results

### 3.1 Metagenomic characterization of the four types of seed *Pei*

Sequencing using the Illumina PE150 platform generated a total of 134.14 Gb of raw sequences. After quality filtration procedures, the dataset was consolidated to 129.05 Gb of high-quality sequences without host data, averaging 32.26 Gb per sample. The ratio of valid data was 96.20%. Among the non-host clean data of all samples, the proportion of base quality greater than Q30 was 93.73%, and the G+C content was 41.21%. The sequencing information of individual samples is shown in Supplementary Table S1. After assembly, a total of 272,988 sequences (length > 500 bp) were obtained, with a sequence average length of 1,194 bp. A total of 176,618 non-redundant ORFs were predicted by MetaGeneMark.

**Figure 1:**
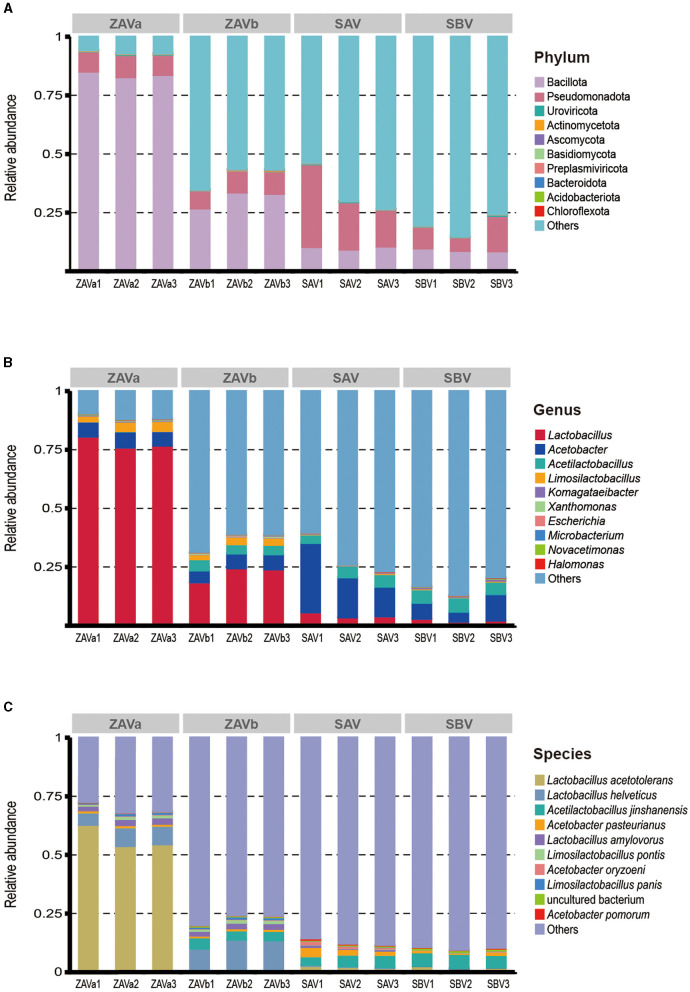
Relative abundance of microorganisms in four groups of seed *Pei* at the phylum **(A)**, genus **(B)**, and species **(C)** levels. Group: ZAVa and ZAVb were seed *Pei* samples from two different manufacturers of Zhenjiang aromatic vinegar (Zhenjiang, China); SAV was seed *Pei* from Shanxi aged vinegar (Taiyuan, China); SBV was seed *Pei* from Sichuan bran vinegar (Meishan, China).

An overview of the taxonomy of the four seed *Pei* samples shows that bacteria were the major taxa (94.48–97.81%, mean 95.94%), followed by viruses (0.35%), eukaryota (0.07%), archaea (0.003%), and others (3.64%). At the phylum level (Figure 1A), the ZAVa was more clearly annotated with only 6.63% of unclassified taxa. The dominant phylum is Bacillota (83.89%), followed by Pseudomonadota (8.90%). As for the other starters, the majority of genes were not annotated to an existing taxonomic phylum (59.41–80.45%). The most dominant phylum in ZAVb, SAV, and SBV was Bacillota or Pseudomonadota. The content of Bacillota in the two samples of ZAV was higher than that in SAV and SBV, while the content of Pseudomonadota in SAV and SBV was higher than that in ZAV. The archaea found in seed *Pei* were Euryarchaeota, “*Candidatus Korarchaeota*”, “*Candidatus Bathyarchaeota*”, “*Candidatus Thorarchaeota*”, and “*Candidatus Woesearchaeota*”. Eukaryota in seed *Pei* samples included Ascomycota, Basidiomycota, Mucoromycota, Microsporidia, and Zoopagomycota. Uroviricota, Preplasmiviricota, Nucleocytoviricota, Cossaviricota, Artverviricota, and Pisuviricota were viral phyla found in seed *Pei*.

The microbiological distribution at the genus level (Figure 1B) showed that a total of 598 genera were detected. The dominant genera (abundance > 1%) in ZAVa were *Lactobacillus* (77.79%), *Acetobacter* (6.5%), and *Limosilactobacillus* (3.6%). The dominant genera (abundance > 1%) in ZAVb major genera were *Lactobacillus* (22.33%), *Acetobacter* (5.94%), and *Limosilactobacillus* (2.82%), which differed from ZAVa by also containing *Acetilactobacillus* (4.28%). The dominant genera (> 1% abundance) of SAV and SBV were similarly composed of *Acetobacter* (19.78%, 7.45%), *Acetilactobacillus* (4.71%, 5.8%), and *Lactobacillus* (4.4%, 2.27%).

At the species level (Figure 1C), we detected a total of 1,839 species. The main species (abundance > 1%) in four samples were either *Acetobacillus* sp. or *Lactobacillus* sp. The dominant species in ZAVa were *Lactobacillus acetotolerans* (57. 07%), *Lactobacillus helveticus* (6.95%), *Lactobacillus amylovorus* (2.43%), and *Limosilactobacillus pontis* (1.06%). There dominant species in ZAVb were *Lactobacillus helveticus* (11. 93%), *Acetilactobacillus jinshanensis* (4.28%), *Lactobacillus amylovorus* (2.29%), and *Limosilactobacillus pontis* (1.25%), with the relative abundances exceeding 1%. The dominant species in SAV were *Acetilactobacillus jinshanensis* (4.71%), *Acetobacter pasteurianus* (2.7%), and *Lactobacillus acetotolerans* (2.2%), with a relative abundance above 1%. In SBV, the dominant species were *Acetilactobacillus jinshanensis* (5.8%) and *Lactobacillus acetotolerans* (2.03%). The four starters differed in the composition of the dominant species. More information on the top 60 annotated species is shown in Supplementary Figure S1.

The composition of the main acid-producing microbial species, *Lactobacillus*, was found to be 54 species (abundance: 67.63%), followed by 48 (15.25%), 45 (3.33%), and 32 (2.08%) in ZAVa, ZAVb, SAV, and SBV, respectively. *Limosilactobacillus* comprised 28 (3.21%), 27 (2.61%), 27 (0.26%), and 32 species (0.26%). *Acetobacter* consisted of 43 (1.93%), 43 (1.79%), 40 (5.56%), and 42 species (2.41%). It is evident that acid-producing species are highly diverse in seed *Pei*.

### 3.2 Differences in microbial communities among the four types of seed *Pei*

**Figure 2:**
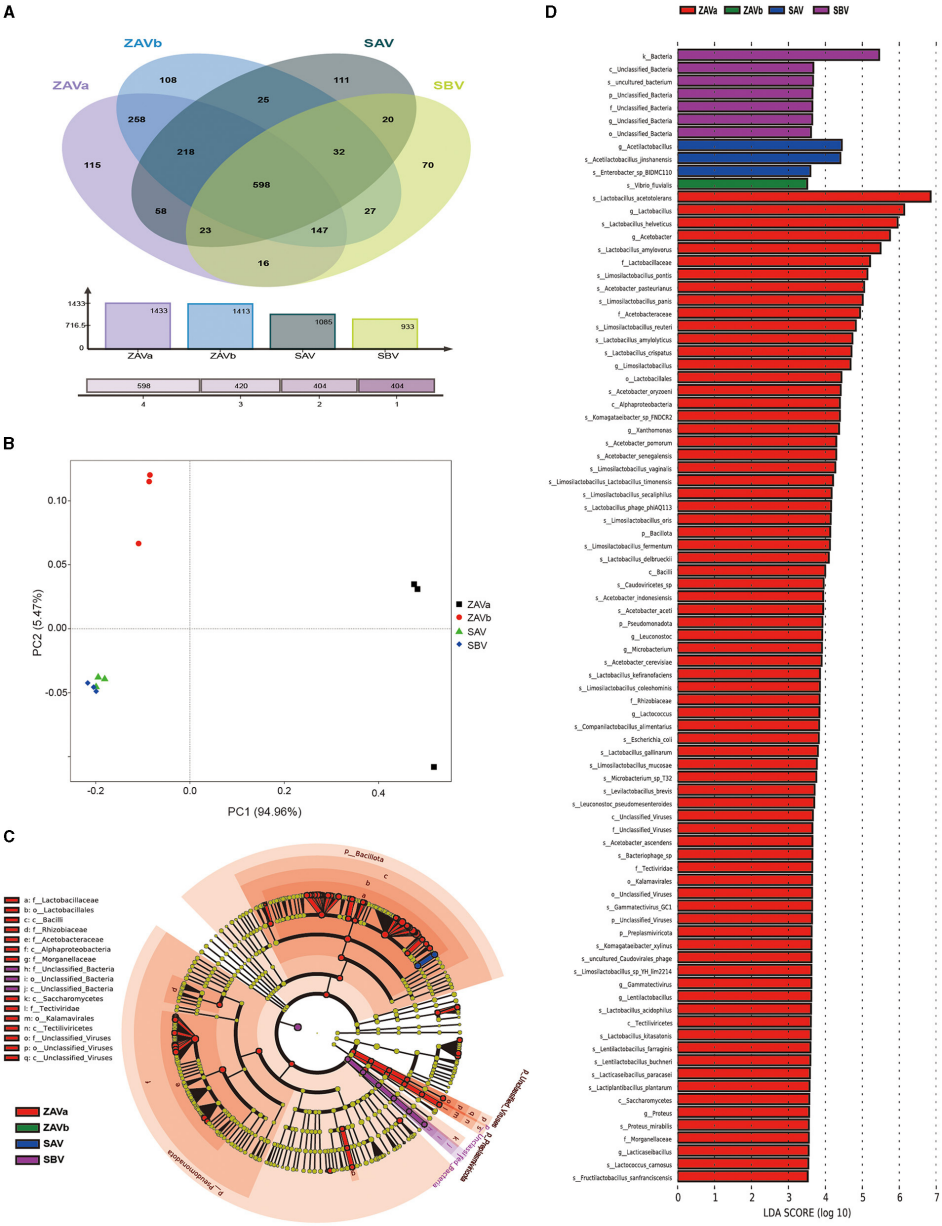
Venn diagram of the distribution of species in four groups of seed *Pei* **(A)**; PCoA plot of the distribution of species in four groups of seed *Pei* **(B)**; differential biomarker analysis in four groups of seed *Pei* by linear discriminant analysis effect size (LEfSe), microbiota cladogram **(C)**; LDA effect size analysis **(D)**.

The Venn diagram (Figure 2A) illustrates the distribution of the four samples in terms of species diversity: ZAVa and ZAVb had a higher number of species, 1,433 and 1,413, respectively, followed by SAV (1,085), and SBV contained the lowest number of species (933). Out of the total 1,839 species, 598 species could be detected in all four types of seed *Pei*. Each *Pei* contained their unique species. ZAVa had the highest number of unique species (115), while SBV had the lowest number of unique species (70).

To obtain an overview of the microbial diversity and richness in the seed *Pei*, we calculated the alpha diversity index (ACE, Chao1, Shannon, and Simpson) (Supplementary Table S2). Further significance analysis (*p* < 0.05) revealed that the ACE and Chao1 indices of ZAVa and ZAVb were significantly higher than those of SBV and SAV. The Shannon and Simpson indices of ZAVa were the highest, followed by those of ZAVb, while the Shannon and Simpson indices of SBV and SAV were the lowest. It can be seen that the diversity and richness of microorganisms in ZAVa and ZAVb species were higher than those in SBV and SAV. The ZAVa species also exhibited the highest degree of dominance.

Beta diversity analysis, specifically the principal coordinates analysis (PCoA), revealed elucidated microbial composition variations among the four seed *Pei* samples (Figure 2B). Principal component 1 accounted for 94.96% of the total community variations, representing the primary explanatory axis, while principal component 2 explained an additional 5.47% variation. The SAV and SBV groups were positioned in close proximity, delineating a separation from the ZAVa and ZAVb groups. This result indicates that, despite differing geographical origins, the seed *Pei* samples from the SAV and SBV groups harbor similar microbial communities. Conversely, the ZAVa and ZAVb groups, although geographically closer, exhibit distinct microbial compositions from each other and also differ from the SAV and SBV groups. Clustering tree analysis of microbiome species based on Bray–Curtis distance also revealed similar sample relationships (Supplementary Figure S2).

LEfSe analysis (Figures 2C, D) revealed the taxon with LDA values > 3.5, indicating the distinctive taxon of seed *Pei*. The indicated species of SBV was an uncultured bacterium, which was significantly different from the species to the kingdom level. There were two distinctive species of SAV: *Acetilactobacillus jinshanensis* and *Enterobacter* sp. BIDMC110. The indicated species of ZAVb is *Vibrio fluvialis*. ZAVa has the highest number of significantly different species, with three different phyla including unclassified viruses, namely, *Preplasmiviricota, Pseudomonadota*, and *Bacillota*. There were 11 different genera in ZAVa: *Acetobacter, Xanthomonas, Gammatectivirus, Microbacterium, Proteus, Limosilactobacillus, Lacticaseibacillus, Lentilactobacillus, Lactobacillus, Leuconostoc*, and *Lactococcus*. At the species level, there were 48 different species, mainly the *Acetobacter* sp. (8), *Limosilactobacillus* sp. (11), and *Lactobacillus* sp. (10).

The microbial co-occurrence network analysis revealed that only six species exhibited specific correlations among different seed *Pei* samples (Supplementary Figure S3). *Acetobacter pasteurianus* showed negative correlations with *Lactobacillus helveticus* and *Limosilactobacillus pontis*, whereas *Lactobacillus amylovorus* exhibited negative correlations with *Acetilactobacillus jinshanensis* and other species. *Acetilactobacillus jinshanensis* displayed positive correlations with other species. These species represent the major functional groups responsible for acid production during vinegar fermentation, and they are primarily negatively correlated.

### 3.3 Investigation of metagenomic function in carbohydrate metabolism and main metabolic pathways of four types of seed *Pei*

**Figure 3:**
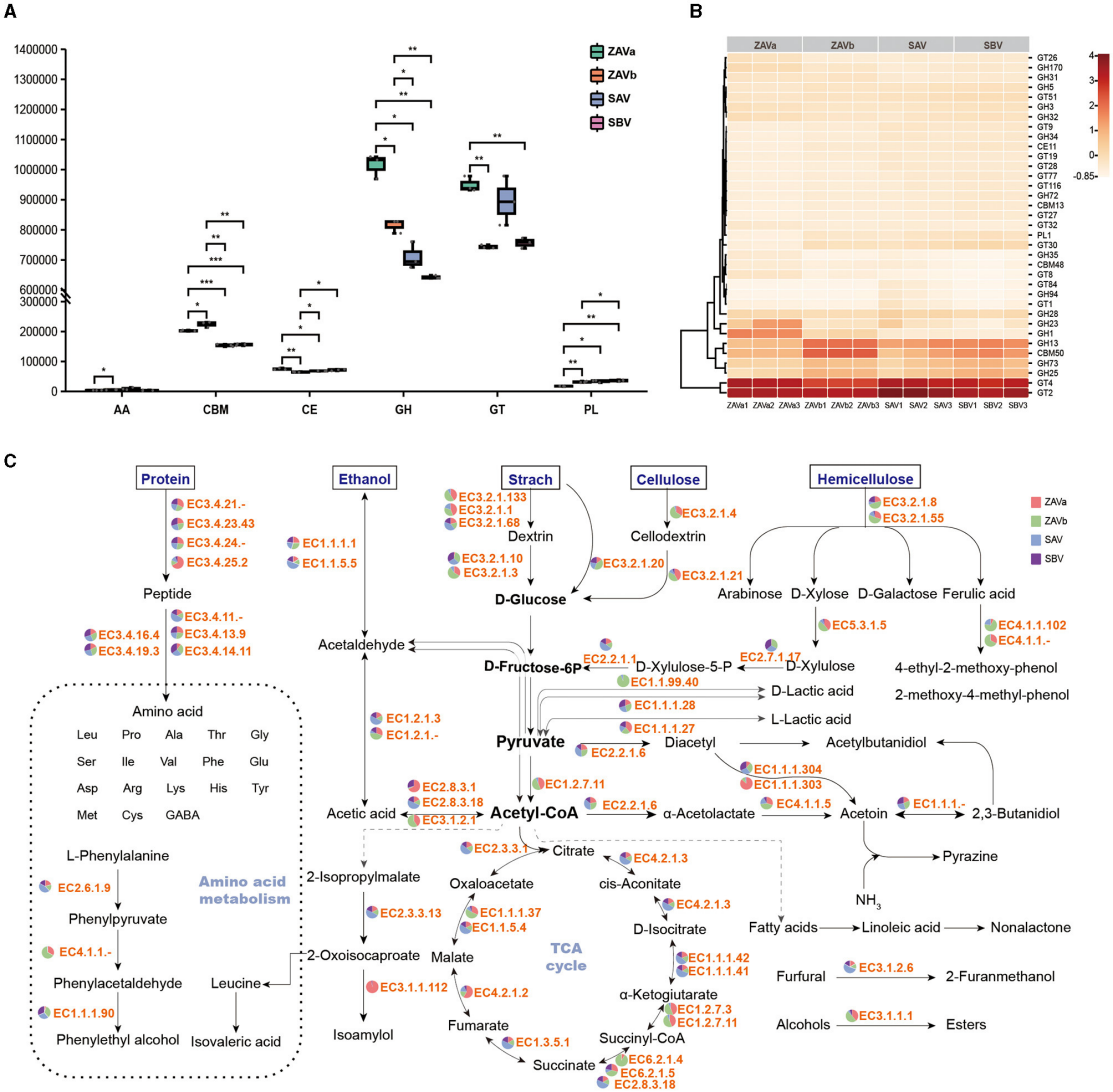
Comparative analysis of carbohydrate active enzyme (CAZy) with different composition and diversity in seed *Pei*. Abundance of *six* CAZy families in four groups of seed *Pei* **(A)**; heatmap plot depicting CAZy family abundance in each sample **(B)**; metabolic networks of major flavor substances in SSF vinegar and KEGG-based enzyme abundance **(C)**. Significant differences are presented by ***p < 0.001, **0.001 ≤ *p* < 0.01, and *0.01 ≤ *p* < 0.05.

Among KEGG gene annotations, carbohydrate metabolism genes were the most abundant in different functional categories (Supplementary Figure S4). The number of genes annotated to the CAZy database accounted for 6.61% of the total number of unigenes. The total number of genes encoding CAZymes in enzyme classes of glycoside hydrolases (GH), glycosyl transferases (GT), carbohydrate-binding modules (CBM), carbohydrate esterases (CE), polysaccharide lyases (PL), and auxiliary activities (AA) was 5,082, 4,810, 1,194, 365, 116, and 107, respectively. The most prevalent identified gene clusters were the GH > GT > CMB > CE > PL > AA families. The differences in the number of the six classes of CAZymes in SAV and SBV were not significant (Figure 3A). There were significant differences in the CAZymes classes between ZAVa and the other samples. Specifically, the relative content of GH, GT, and CE was significantly high, while the relative content of AA and PL was low in all four samples. Additionally, the number of genes in the CBM family in ZAVa was lower than that in ZAVb but higher than that in SAV and SBV.

The heatmap (Figure 3B) of the carbohydrate-active enzyme classes showed that GT2 and GT4 were most prevalent in seed *Pei*. These genes are involved in the formation of polysaccharides in the cell walls of various organisms (Howe et al., 2016) as well as in cellulose synthesis (Stone et al., 2010). The next highest content was of GH13, also known as the α-amylase family, which is one of the largest GH families in CAZy. It is the predominant functional enzyme in the hydrolysis of starch feedstocks (Møller et al., 2016). The content of the GH13 family varied in the four seed *Pei* samples, with the highest content found in ZAVb, followed by SBV. GH23, a peptidoglycan lyase of bacterial and phage origin, and GH1, a peptidoglycan lyase containing a variety of β-glycosidases, were found in higher concentrations in the ZAVa samples than in the other samples. The CBM family with the highest concentration in seed *Pei* was CBM50, which consists of modules that bind to peptidoglycan and chitin (Andrade et al., 2017). The PL family with the highest concentration was PL1, but the PL family was not prevalent in seed *Pei*. PLs use an elimination mechanism to cleave glycosidic bonds of complex carbohydrates, and PL1 mainly acts in the hydrolysis of pectin. From the analysis of carbohydrate-active enzymes, it can be seen that different sources of seed *Pei* have different carbohydrate enzymes to adapt to different carbohydrate raw materials.

We constructed a metabolic network of the main flavor-producing species in vinegar and compared the proportional relationships of functional genes in the pathway of flavor compound formation across different sample groups (Figure 3C, Supplementary Table S3). Through intergroup comparisons, significant disparities were observed in the absolute levels of genes encoding starch hydrolases, cellulose hydrolases, and hemicellulose hydrolases within the diverse seed *Pei* samples. Notably, the ZAVb sample exhibited the highest expression of these three enzyme genes, indicating a superior capability for efficient grain substrate utilization. This is achieved by effectively converting starch, cellulose, and hemicellulose into pentose and hexose sugars, which serve as a readily accessible carbon source for a wide range of microorganisms. When it comes to the hydrolysis of proteins into free amino acids, the majority of enzyme genes demonstrated comparable distributions across the different seed *Pei* samples, except for the increased levels of EC 3.4.14.11 in ZAVb and SBV samples, the elevated prevalence of EC 3.4.25.2 in ZAVa, and the highest content of EC 3.4.11 found in SAV. Functionally, EC 3.4.14.11 corresponds to Xaa-Pro dipeptidyl-peptidase, EC 3.4.25.2 corresponds to ATP-dependent peptidase, and EC 3.4.11. encompasses aminopeptidases.

### 3.4 Correlation between the microbiota and physicochemical properties

**Figure 4:**
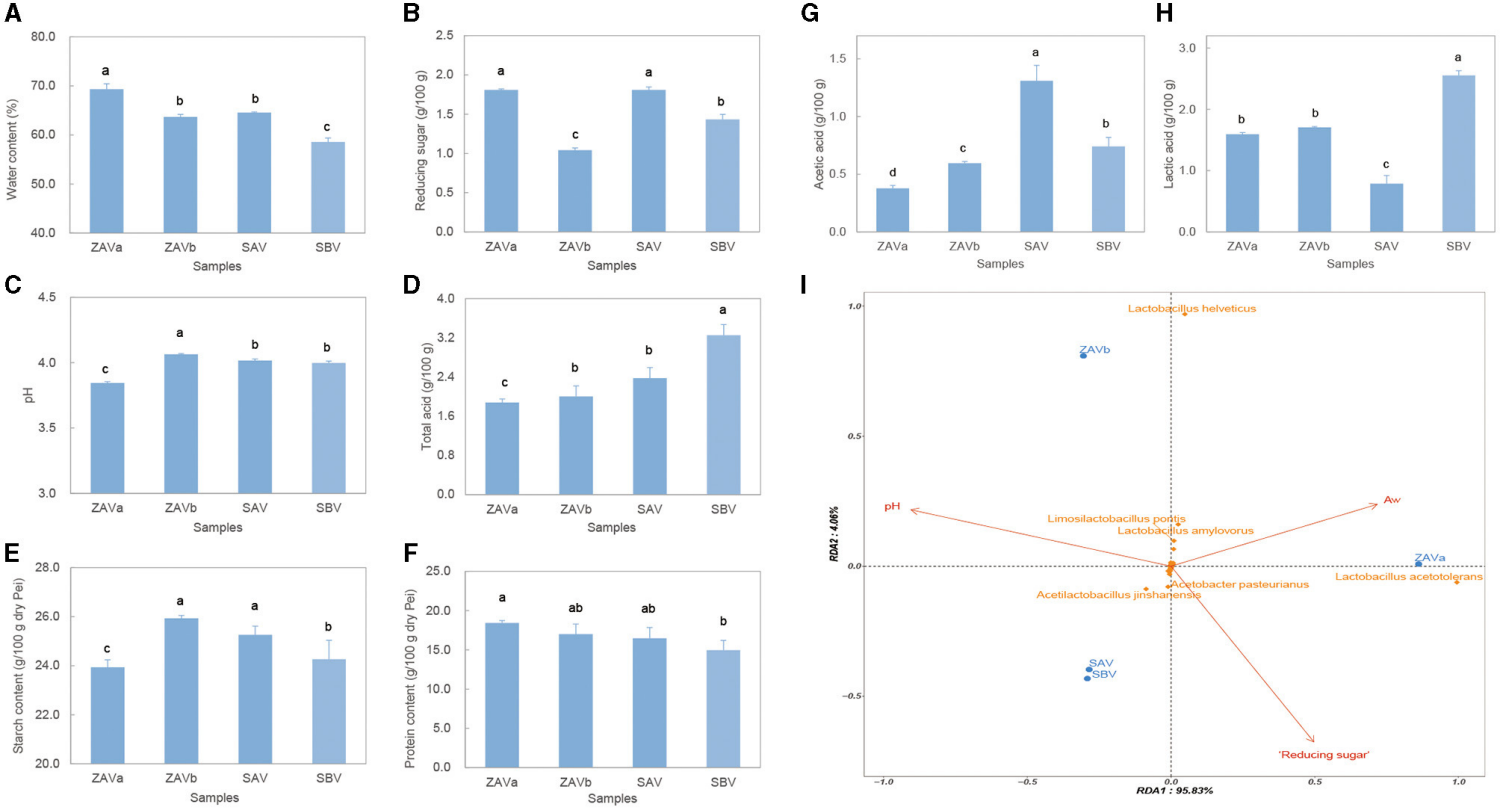
Physicochemical properties of seed *Pei*, water content **(A)**; reducing sugar content **(B)**, pH **(C)**; total acid content **(D)**; total starch content of dry *Pei* **(E)**; total protein content of dry *Pei* **(F)**; acetic acid content **(G)**; lactic acid content **(H)**; RDA of main microorganisms and physicochemical properties for different seed *Pei* **(I)**. Columns in **(A–H)** relied on the replicates of three experiments, columns with different superscript letters (a–d) are significantly different (*p* < 0.05).

The water content, reducing sugar content, pH value, total acid content, acetic acid content, lactic acid content, total starch content, and total protein content of the *Pei* were measured (Figures 4A–H). There were significant differences (*p* < 0.05 in all cases) in the physicochemical properties of four seed *Pei* samples, with protein content showing the least variation among the aforementioned physicochemical parameters. ZAVa had the highest moisture content, lowest pH, lowest total acid, lowest acetic acid, and lowest starch content and also contained the highest reducing sugar content. ZAVb and SAV did not show significant differences in moisture, total acid, starch, and protein content but differed in reducing sugar, acetic acid, lactic acid content, and pH. SBV had the lowest moisture, the highest total acid, and the highest lactic acid content. Moreover, we conducted RDA on the relationship between the physicochemical properties and microbial communities at the species level (abundance > 0.1%) (Figure 4I). We found that three out of the eight environmental factors (Aw, pH, and reducing sugar) explained 99.89% of the variation in the microbial community structure. Among these factors, the reducing sugar content had the greatest impact on the microbial community structure, followed by pH value and then moisture content. It is noteworthy that the reduction in sugar content had a smaller impact on the ZAVb community compared to other types of *Pei*. In contrast, water content had the most significant effect on the ZAVa community, while pH value had the greatest impact on the ZAVb community. The RDA revealed that two species of lactic acid bacteria showed the most significant response to the environmental factors. Specifically, *Lactobacillus acetotolerans* was greatly influenced by Aw and reducing sugar content, while *Lactobacillus helveticus* was more sensitive to Aw and pH value.

### 3.5 MAGs in seed *Pei*

**Figure 5:**
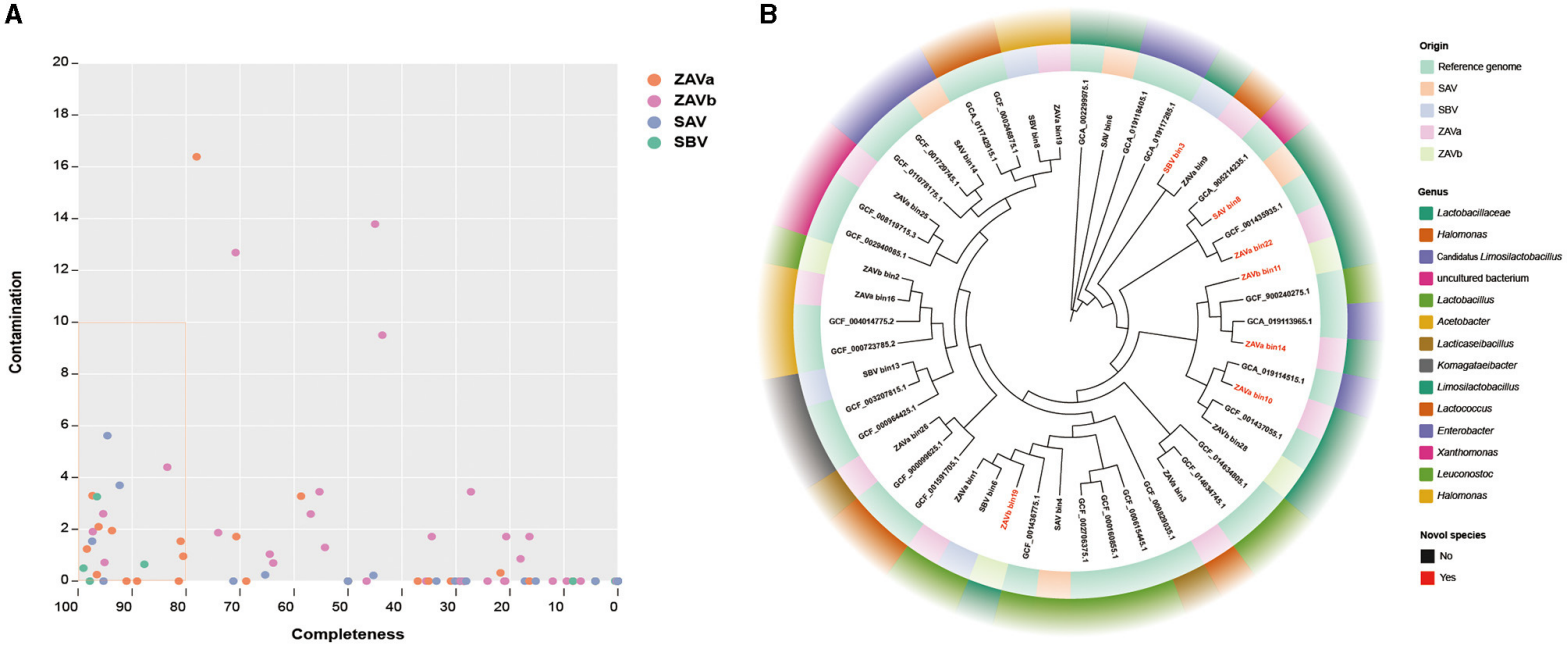
The assembly and analysis of metagenome-assembly genomes (MAGs) of four groups of seed *Pei*. MAG quality assessment by CheckM **(A)**. Points within the orange boxed line represent high-quality MAGs (≥ 80% completeness and ≤ 10% contamination). The phylogenetic tree based on 120 bacterial marker genes of high-quality MAGs and reference genomes **(B)**.

A total of 98 MAGs were identified from the metagenomes of individual groups with 27 MAGs in ZAVa, 36 MAGs in ZAVb, 21 MAGs in SAV, and 14 MAGs in SBV. The MAGs obtained from the bins were checked for genomic quality, and 22 high-quality MAGs (≥ 80% completeness and ≤ 10% contamination) were selected for subsequent analysis (Figure 5A). A total of 22 high-quality MAGs (ZAVa 10, ZAVb 4, SAV 4, and SBV 4) were taxonomically assigned to two phyla, three classes, five orders, six families, and nine genera. The results showed *Limosilactobacillus* had the highest number of MAGs in the high-quality MAGs dataset for all samples, followed by *Lactococcus* (Figure 5B). A total of seven MAGs were considered potentially novel species.

## 4 Discussion

The acetic acid fermentation process in SSF of vinegar mainly involves the microbial conversion of ethanol, bran, and other raw materials into acetic acid, lactic acid, and other flavor compounds. In this process, the fermentation starter is one of the main sources of microorganisms (Huang et al., 2022) that significantly influences the fermentation process, the extent of raw material utilization, and the flavor of the final product. Studying the microbial community of vinegar fermentation starters in different regions helps us understand the fundamental reasons for variations in fermentation and flavor and provides insights for constructing artificial fermentation starters.

This study represents the first comparative analysis of microbial species composition, functional genes, and metabolic pathways in different SSF seed *Pei* samples. A total of 598 genera and 1,893 species were identified, surpassing the number of species and genera obtained through amplicon sequencing. Bacteria (> 90%) were found to be the dominant microbial group in the seed *Pei*, consistent with previous research (Wu et al., 2017; Li et al., 2023), indicating their significant contribution during acetic acid fermentation. Compared to previous metagenomic studies (Wu et al., 2017), this study elucidated the annotation of viral groups, revealing their presence in the seed *Pei* and higher abundance compared to eukaryotes and archaea. In a specialized virome study of the fermentation process of ZAV, it was found that viruses are rich in carbohydrate metabolism genes that may be involved in the bacterial fermentation process (Yu et al., 2022). This finding suggests that the presence of viruses (bacteriophages) may have an impact on vinegar fermentation. The samples ZAVb, SAV, and SBV also contained numerous unclassified groups, indicating the presence of “microbial dark matter” that has not yet been isolated, cultured, or characterized genomically. Further research on these “dark matter” organisms is essential to unravel the fermentation mechanisms of vinegar. While the main microbial species composition of the seed *Pei* observed in this study aligns with previous findings (Nie et al., 2017; Huang et al., 2022; Li et al., 2023, 2024), consisting primarily of acetic acid bacteria and lactic acid bacteria such as *Lactobacillus, Acetobacter, Limosilactobacillus*, and *Acetilactobacillus*, the specific species composition and abundance vary among different regions.

A previous study identified the genera of *Acetobacter, Lactobacillus, Enhydrobacter, Lactococcus, Gluconacetobacter, Bacillus*, and *Staphylococcus* as crucial microbiota contributing significantly to the synthesis of flavor components of ZAV. Their prevalence and functional roles were outlined based on amplicon sequencing (Wang et al., 2016). However, in the four seed *Pei* samples we collected, *Enhydrobacter* and *Gluconacetobacter* were not detected, while *Bacillus* (> 0.005%), *Staphylococcus* (> 0.02%), and *Lactococcus* (> 0.1%) were found to be present but in low abundance. Another study identified 11 core functional microorganisms in Sichuan Shai vinegar using PacBio sequencing combined with the traditional culture method, namely *Brettanomyces bruxellensis, Pichia kudriavzevii, Acetobacter pasteurianus, Acetobacter pomorums, Lactobacillus acetotolerans, Lactobacillus amylolyticus, Lactobacillus amylovorus, Lactobacillus fermentum, Lactobacillus plantarum, Clostridium beijerinckii*, and *Lichtheimia ramose* (Li et al., 2024). The core functional microorganisms identified in this study were also detected in our seed *Pei*, and the effects of the acid-producing microorganisms were consistent with our results. However, *Brettanomyces bruxellensis, Pichia kudriavzevii, Clostridium beijerinckii*, and *Lichtheimia ramose* were present in low abundance. According to previous studies, some low-abundance taxa in the seed *Pei* were associated with certain flavor compounds in vinegar. For example, *Bacillus amyloliquefaciens* was found to enhance acetoin and tetramethylpyrazine contents when added to *Daqu* (Zhang et al., 2017). Additionally, *Pantoea, Pediococcus*, and *Rhizobium* were found to be associated with esters, particularly propanoic acid-2-hydroxy-ethyl ester, hexanoic acid ethyl ester, and ethyl acetate in SAV (Zhu et al., 2018). Theoretically, these low-abundance taxa may play essential roles in vinegar fermentation, but their specific functions and mechanisms require further investigation.

Furthermore, through LEfSe analysis, we identified characteristic species for each vinegar starter. ZAVa was found to harbor the highest number of characteristic species, including both core and non-core species. Two of the seed *Pei* pairs share similar raw materials, production processes, and geographical proximity. However, compared to seed *Pei* with different raw materials, production processes, and geographical distributions, the differences in microbial community structure and functionality are more noticeable within these pairs (as evidenced by PCoA). Conversely, the other two seed *Pei* samples, with distinct raw materials, processes, and geographical distributions, exhibit more similar species compositions. Therefore, we speculate that the long-term cultivation and preservation practices of unique microbial strains by individual manufacturers are the main factors contributing to microbial community differences in the SSF of vinegar.

In the co-occurrence network analysis, due to significant microbial community variations among the seed *Pei* samples, we only observed correlations between five core microorganisms involved in vinegar fermentation. This finding indicates a negative correlation between the key acid-producing microorganisms, such as *Acetobacter* and *Lactobacillus*, which aligns with previous studies on vinegar microbiota (Hutchinson et al., 2019; Chai et al., 2020; Xia et al., 2022). Furthermore, our review of previous research reveals a consistent trend of competitive dynamics between *Acetobacter* and *Lactobacillus* in different vinegar production processes (Wang et al., 2016; Nie et al., 2017; Huang et al., 2022; Li et al., 2023). In some instances, the abundance of *Acetobacter* decreased with the fermentation process and the abundance of *Lactobacillus* gradually increased (Huang et al., 2022), while in other cases, the opposite scenario is observed (Wang et al., 2016; Nie et al., 2017; Li et al., 2023). Therefore, the distinct composition and ratio of *Acetobacter* and *Lactobacillus* in fermentation starters can support successful vinegar fermentation. However, the specific impacts of these differences on the vinegar fermentation process and resulting flavors require further investigation. The observed negative correlations among the five microorganism groups may be attributed to interspecies competition or environmental factors driving species succession.

Functionality-wise, the most abundant genes were the GH and GT family of carbohydrate metabolism. These abundant genes have also been observed in fermented vegetables and dairy products (Liang et al., 2023; Liu et al., 2023). When compared to other seed *Pei* samples, ZAVa displayed the highest abundance of GH1 genes associated with cellulose degradation, indicating that it may be more effective in breaking down the cellulosic component of lignocellulose. ZAVb exhibited a higher abundance of GH13 family genes associated with starch hydrolysis, suggesting a possible benefit in raw material usage. The gene abundance of GT2 and GT4 was highest in SAV. These findings suggest that the seed *Pei* has a wide variety of carbohydrate-active enzymes that are associated with the rapid utilization of various carbohydrates to start fermentation and provide unique flavor characteristics. Based on KEGG annotation of the main fermentation pathways, the distribution of genes involved in acetic acid production was relatively balanced among the four starters. Notably, ZAVb possessed a higher abundance of genes involved in the decomposition of macromolecular substrates such as starch, cellulose, and hemicellulose. Furthermore, throughout this study, different seed *Pei* samples exhibited variations in the primary enzyme systems responsible for protein degradation during vinegar fermentation.

The RDA was conducted to reveal the correlations between physicochemical factors and microbial distribution in the seed *Pei*. The factors such as water content, reduced sugar content, and pH value were found to have a significant impact on the distribution of key microbial groups. However, further experimental verification is required to determine the actual effects. This result suggests that controlling water content, pH, and reducing sugar content is crucial for ensuring successful vinegar fermentation.

## 5 Conclusion

In this study, we focused on investigating the microbial distribution and functionality of seed *Pei*, which serve as fermentation starters obtained from four manufacturers in three provinces. The dominant microbial group in the seed *Pei*, according to metagenomic sequencing, was bacteria, followed by viruses, eukaryotes, and archaea. Variations in microbial communities were observed among seed *Pei* from different manufacturers, with *Lactobacillus* sp. and *Acetobacter* sp. being the main functional groups. However, specific species composition and abundance varied. Seed *Pei* from SAV and SBV showed a higher similarity in microbial composition, while significant differences were observed when compared to the two forms of ZAV. The distribution of functional genes revealed a similar pattern to the species composition, with SAV and SBV exhibiting more similarity in functional gene composition than the two forms of ZAV. These findings suggest that the differences in microbial composition and functionality among seed *Pei* are not only geographically driven but are also related to the long-term cultivation and preservation practices of microbial strains by different manufacturers. Additionally, RDA of physicochemical factors and microbial communities revealed that water content, pH, and reducing sugar content are significant factors influencing microbial distribution. Furthermore, we also identified seven MAGs that could potentially represent novel species. This study contributes to our understanding of the properties and functionalities of fermentation starters used in SSF vinegar fermentation, providing insights into the utilization or modification of microbial compositions to optimize vinegar fermentation processes.

## Data availability statement

The datasets presented in this study can be found in online repositories. The names of the repository/repositories and accession number(s) can be found at: https://db.cngb.org/cnsa/, CNP0005244.

## Author contributions

DH: Conceptualization, Funding acquisition, Writing – original draft. YYa: Data curation, Methodology, Writing – original draft. ZG: Data curation, Formal analysis, Writing – original draft. KC: Data curation, Formal analysis, Writing – original draft. SD: Formal analysis, Visualization, Writing – original draft. YZ: Investigation, Methodology, Writing – original draft. YW: Writing – review & editing. ZY: Visualization, Writing – original draft. KW: Writing – review & editing. PL: Software, Writing – original draft. CR: Writing – review & editing. YYu: Funding acquisition, Project administration, Writing – review & editing.
