# Supplementary material for: Metagenomics profiling of the microbial community and functional differences in solid-state fermentation vinegar starter (seed Pei) from different Chinese regions
Source: Front Microbiol. 2024 May 2;15:1389737. doi: 10.3389/fmicb.2024.1389737 (PMC11096547; doi:10.3389/fmicb.2024.1389737)
Supplement: Supplementary file 1 [file Data_Sheet_1.docx]

Supplementary Material

**Supplementary Table 1.** Summary information of metagenomics sequencing.

| Sample | Raw Data (Gbp) | Clean Data (Gbp) | Clean Data Q30 (%) | Clean Data GC (%) | Effective (%) |
| --- | --- | --- | --- | --- | --- |
| ZAVa | 11.14±0.14 | 11.11±0.14 | 93.38±0.11 | 39.54±0.31 | 99.73±0.05 |
| ZAVb | 11.39±0.38 | 11.33±0.37 | 93.97±0.03 | 41.63±0.37 | 99.48±0.10 |
| SAV | 11.17±0.39 | 11.14±0.43 | 93.8±0.43 | 42.61±1.26 | 99.7±0.17 |
| SBV | 11.01±0.11 | 10.98±0.12 | 93.77±0.66 | 41.05±0.47 | 99.7±0.05 |

**Supplementary Table 2.** Alpha diversity index of microbial community in four seed *Pei*.

| Sample | ACE | Chao1 | Shannon | Simpson |
| --- | --- | --- | --- | --- |
| ZAVa | 1274.1±25.1^a^ | 1270.0±27.1^a^ | 2.11±0.19^a^ | 0.60±0.05^a^ |
| ZAVb | 1302.3±37.3^a^ | 1299.2±35.6^a^ | 1.68±0.16^b^ | 0.43±0.04^b^ |
| SAV | 784.0±173.0^b^ | 784.5±175.5^b^ | 1.22±0.18^c^ | 0.28±0.04^c^ |
| SBV | 823.7±13.0^b^ | 822.1±14.0^b^ | 0.97±0.16^c^ | 0.23±0.03^c^ |

*p*-value < 0.05.

**Supplementary Table 3.** Enzyme abundance about the formation of major flavor in solid-state fermented vinegar based on KEGG.

| EC number | ZAVa | ZAVb | SAV | SBV |
| --- | --- | --- | --- | --- |
| 1.1.1.- | 34210.63 | 48690.11 | 50371.76 | 50686.37 |
| 1.1.1.1 | 24598.94 | 28670.05 | 22247.69 | 21928.53 |
| 1.1.1.27 | 60022.75 | 35813.33 | 28380.83 | 29926.64 |
| 1.1.1.28 | 37386.11 | 52212.54 | 48704.69 | 58070.02 |
| 1.1.1.303 | 1018.454 | 117.0294 | 33.36456 | 12.48377 |
| 1.1.1.304 | 9735.316 | 24427.69 | 24261.79 | 27415.22 |
| 1.1.1.37 | 94.80968 | 156.9211 | 31.48971 | 16.59655 |
| 1.1.1.41 | 1031.256 | 901.9002 | 3273.371 | 1038.449 |
| 1.1.1.42 | 763.1202 | 705.7756 | 1780.785 | 650.9171 |
| 1.1.1.90 | 2051.541 | 13855.4 | 12464.17 | 13127.4 |
| 1.1.5.4 | 1785.103 | 1523.698 | 5060.117 | 1556.76 |
| 1.1.5.5 | 1691.965 | 1527.199 | 5184.48 | 2020.597 |
| 1.1.99.40 | 0 | 14.61195 | 1.018245 | 0 |
| 1.2.1.- | 228.1679 | 335.6073 | 57.65763 | 122.858 |
| 1.2.1.3 | 1358.549 | 1176.494 | 3903.288 | 1332.995 |
| 1.2.7.11 | 6.404465 | 7.16129 | 0.950516 | 0 |
| 1.2.7.11 | 6.404465 | 7.16129 | 0.950516 | 0 |
| 1.2.7.3 | 6.404465 | 7.16129 | 0.950516 | 0 |
| 1.3.5.1 | 2645.926 | 2567.915 | 8041.082 | 2948.716 |
| 2.2.1.1 | 4581.414 | 3853.937 | 11553.54 | 4087.751 |
| 2.2.1.6 | 5301.014 | 6329.13 | 8761.645 | 3305.57 |
| 2.3.3.1 | 1221.474 | 1121.943 | 3244.001 | 950.5863 |
| 2.3.3.13 | 1284.26 | 1161.02 | 3518.364 | 1337.127 |
| 2.6.1.9 | 1043.845 | 878.1108 | 2286.507 | 867.1524 |
| 2.7.1.17 | 556.5918 | 12751.63 | 12096.18 | 14347.15 |
| 2.8.3.1 | 197.4198 | 7.806797 | 3.789497 | 87.0897 |
| 2.8.3.18 | 705.0964 | 616.2737 | 2055.762 | 759.1872 |
| 2.8.3.18 | 705.0964 | 616.2737 | 2055.762 | 759.1872 |
| 3.1.1.1 | 398.7384 | 505.1943 | 50.97538 | 65.04147 |
| 3.1.1.112 | 21.69641 | 0 | 0 | 0.487619 |
| 3.1.2.1 | 3.685573 | 5.320015 | 0 | 0 |
| 3.1.2.6 | 1881.764 | 1631.48 | 6157.729 | 1925.86 |
| 3.2.1.1 | 901.5351 | 728.7914 | 386.4844 | 19.46945 |
| 3.2.1.10 | 1142.738 | 11454.26 | 10193.63 | 11451.12 |
| 3.2.1.133 | 4713.73 | 5681.183 | 894.3532 | 73.13695 |
| 3.2.1.20 | 9592.107 | 26495.61 | 13759.16 | 14298.25 |
| 3.2.1.21 | 1346.902 | 1328.357 | 367.27 | 183.2424 |
| 3.2.1.3 | 65.357 | 107.4561 | 19.286 | 0 |
| 3.2.1.4 | 6100.718 | 10775.84 | 730.186 | 184.2182 |
| 3.2.1.55 | 127.3631 | 217.5216 | 60.17537 | 17.51403 |
| 3.2.1.68 | 2379.198 | 2114.495 | 4709.388 | 2211.034 |
| 3.2.1.8 | 17.6509 | 38.73216 | 6.705088 | 18.17378 |
| 3.4.11.- | 1172.565 | 1015.07 | 3330.802 | 1172.782 |
| 3.4.13.9 | 15939.32 | 16023.57 | 13288.89 | 15705.44 |
| 3.4.14.11 | 16622.38 | 44555.15 | 43553.69 | 56106.36 |
| 3.4.16.4 | 62221.94 | 90471.5 | 94027.67 | 103753.6 |
| 3.4.19.3 | 18738.49 | 30114.5 | 27988.04 | 30433.1 |
| 3.4.21.- | 178980.2 | 123035.6 | 121950.6 | 123020 |
| 3.4.22.70 | 4239.791 | 20649.35 | 17960.35 | 23313.53 |
| 3.4.23.43 | 16630.76 | 17973.42 | 17428.47 | 21680.75 |
| 3.4.24.- | 95758.39 | 87329.51 | 83059.94 | 84888.76 |
| 3.4.25.2 | 18447.12 | 3730.632 | 3705.067 | 1586.338 |
| 4.1.1.- | 1507.947 | 2950.384 | 102.0109 | 0 |
| 4.1.1.- | 1507.947 | 2950.384 | 102.0109 | 0 |
| 4.1.1.102 | 1.045017 | 9.327404 | 3.04278 | 0 |
| 4.1.1.5 | 2784.778 | 3912.995 | 1829.262 | 712.3946 |
| 4.2.1.2 | 15007.13 | 4499.972 | 4416.667 | 1737.449 |
| 4.2.1.3 | 3029.994 | 2734.248 | 8531.357 | 3011.949 |
| 5.3.1.5 | 366.1381 | 430.7941 | 97.57535 | 33.35009 |
| 6.2.1.4 | 1.056254 | 10.62373 | 0 | 0 |
| 6.2.1.5 | 178.8803 | 235.2326 | 61.69122 | 131.6224 |


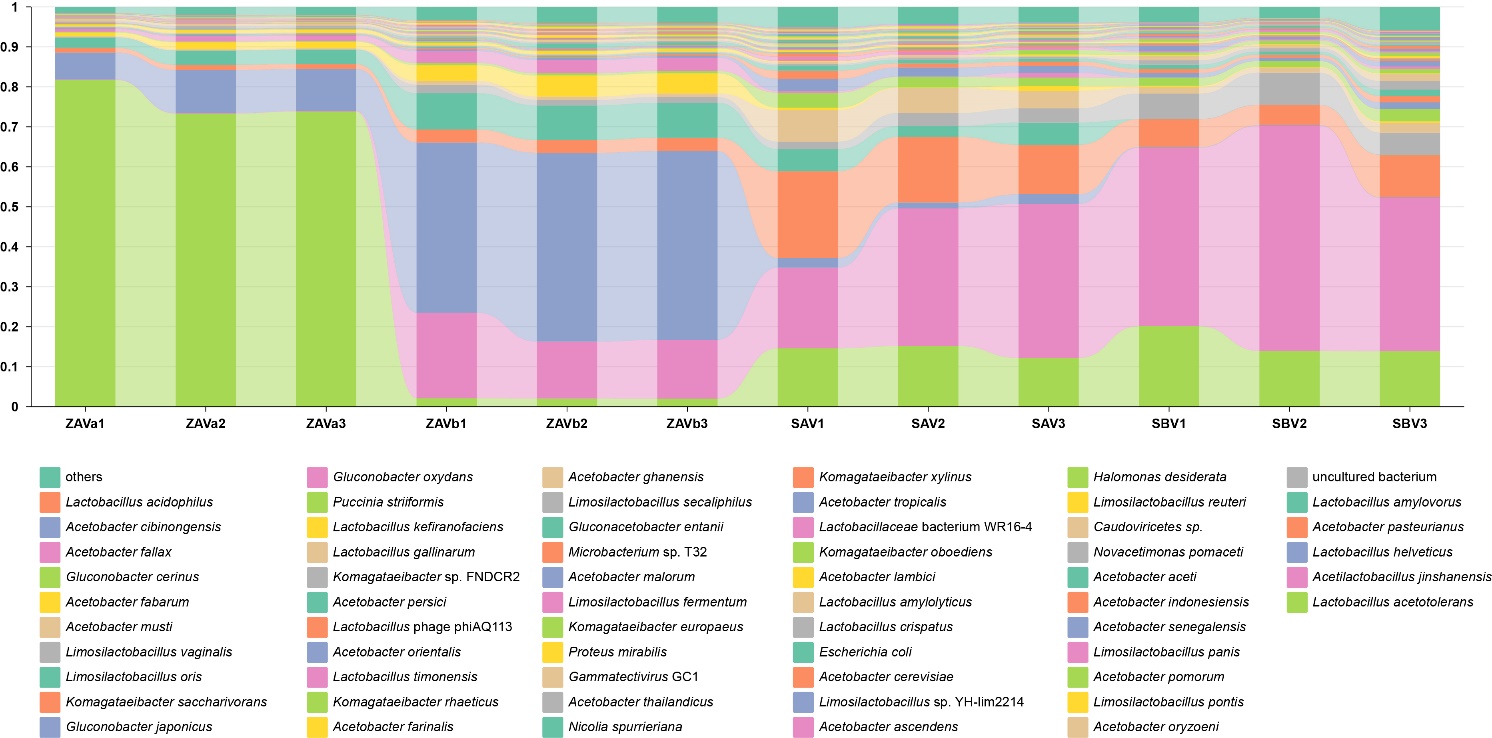


**Supplementary Figure 1.** The distribution of the top 60 species in four seed *Pei*, including only those that have been validly annotated.


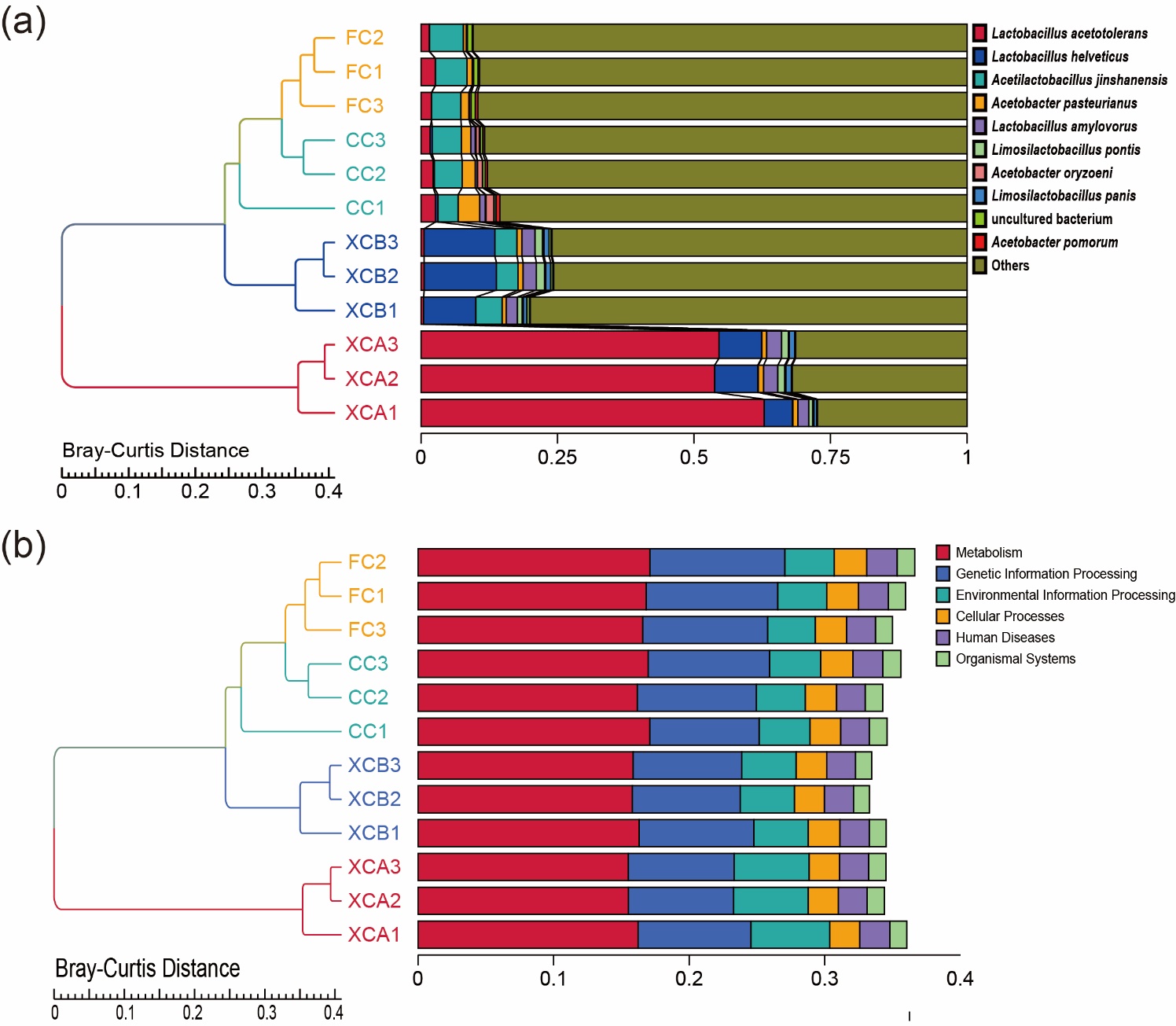


**Supplementary Figure 2.** Clustering tree based on Bray-Curtis distance, clustering and distribution of each sample at species level (a), clustering and distribution of functional genes on KEGG level 1 (b).


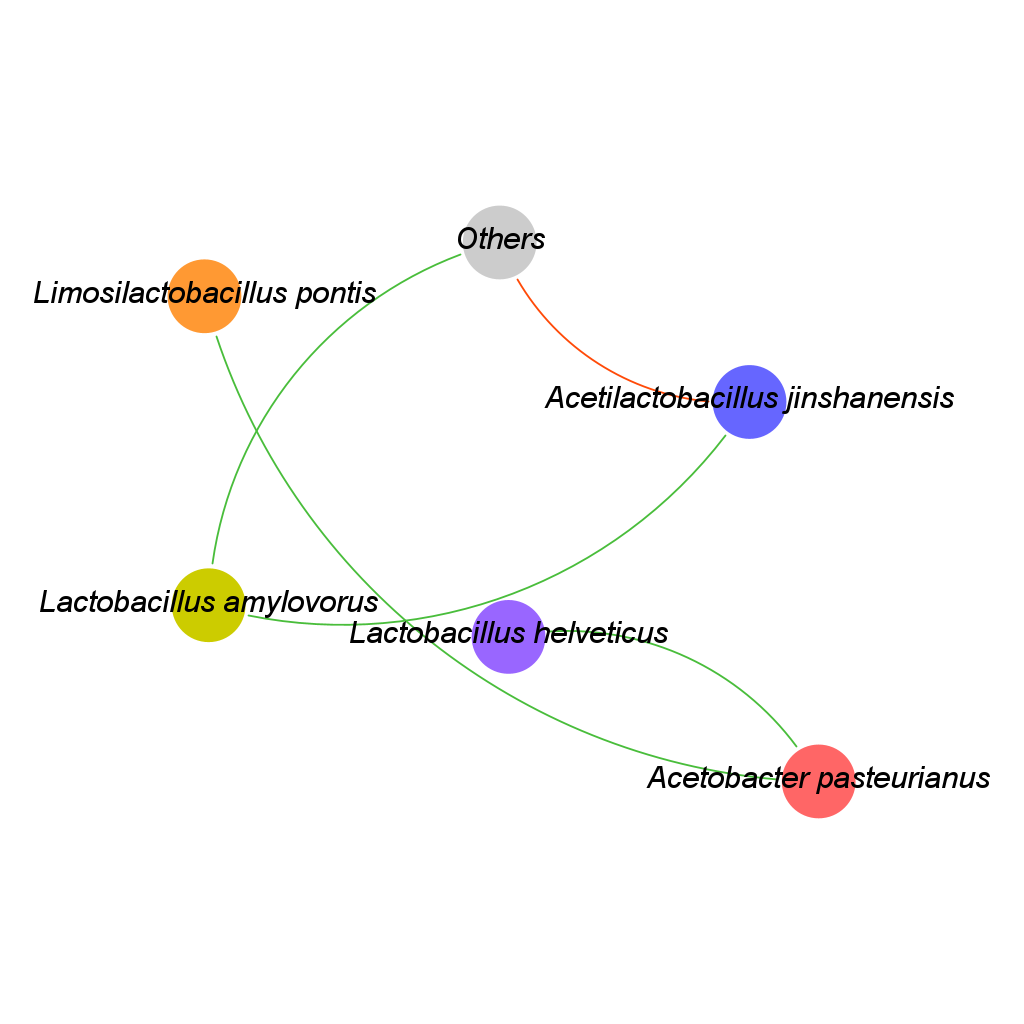


**Supplementary Figure 3.** The co-occurrence network of the seed *Pei* microbiota.


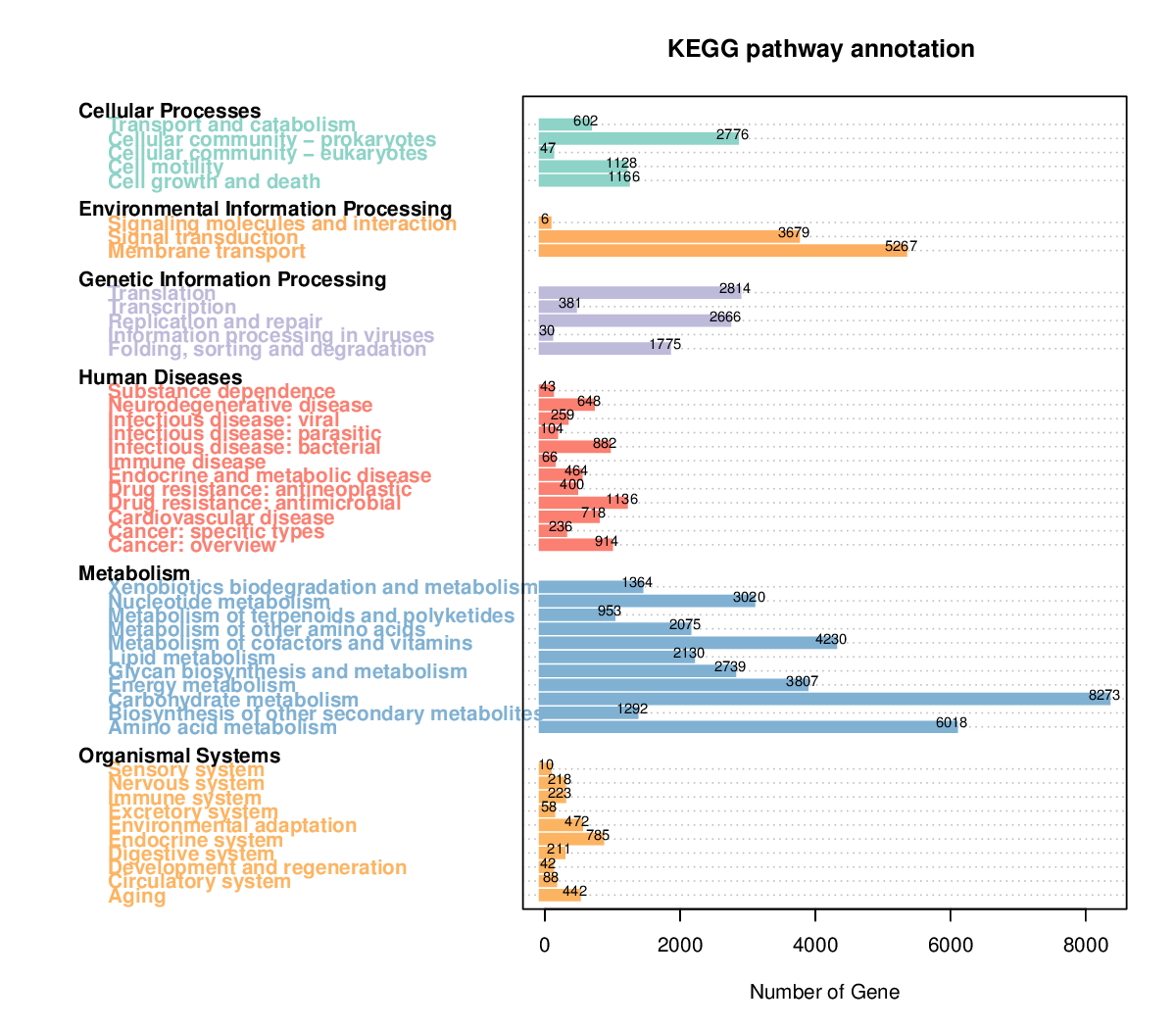
**Supplementary Figure 4.** Functional genes of seed *Pei* base on KEGG database.
